# Low dose tubulin-binding drugs rescue peroxisome trafficking deficit in patient-derived stem cells in Hereditary Spastic Paraplegia

**DOI:** 10.1242/bio.20147641

**Published:** 2014-05-23

**Authors:** Yongjun Fan, Gautam Wali, Ratneswary Sutharsan, Bernadette Bellette, Denis I. Crane, Carolyn M. Sue, Alan Mackay-Sim

**Affiliations:** 1 National Centre for Adult Stem Cell Research, Eskitis Institute for Drug Discovery, Griffith University, Brisbane, QLD 4111, Australia; 2 Kolling Institute for Medical Research, University of Sydney, Sydney, NSW 2065, Australia

**Keywords:** Patient-derived stem cells, Hereditary Spastic Paraplegia, Spastin, Microtubules, Peroxisome trafficking

## Abstract

Hereditary Spastic Paraplegia (HSP) is a genetically heterogeneous group of disorders, diagnosed by progressive gait disturbances with muscle weakness and spasticity, for which there are no treatments targeted at the underlying pathophysiology. Mutations in spastin are a common cause of HSP. Spastin is a microtubule-severing protein whose mutation in mouse causes defective axonal transport. In human patient-derived olfactory neurosphere-derived (ONS) cells, spastin mutations lead to lower levels of acetylated α-tubulin, a marker of stabilised microtubules, and to slower speed of peroxisome trafficking. Here we screened multiple concentrations of four tubulin-binding drugs for their ability to rescue levels of acetylated α-tubulin in patient-derived ONS cells. Drug doses that restored acetylated α-tubulin to levels in control-derived ONS cells were then selected for their ability to rescue peroxisome trafficking deficits. Automated microscopic screening identified very low doses of the four drugs (0.5 nM taxol, 0.5 nM vinblastine, 2 nM epothilone D, 10 µM noscapine) that rescued acetylated α-tubulin in patient-derived ONS cells. These same doses rescued peroxisome trafficking deficits, restoring peroxisome speeds to untreated control cell levels. These results demonstrate a novel approach for drug screening based on high throughput automated microscopy for acetylated α-tubulin followed by functional validation of microtubule-based peroxisome transport. From a clinical perspective, all the drugs tested are used clinically, but at much higher doses. Importantly, epothilone D and noscapine can enter the central nervous system, making them potential candidates for future clinical trials.

## INTRODUCTION

Drug discovery for many neurological disorders is hampered by the lack of models to investigate aetiology and to identify candidate drugs. This problem is potentially greater for rare disorders with genetic heterogeneity such as Hereditary Spastic Paraplegia (HSP). Low prevalence does not provide a strong commercial impetus for drug development and genetic heterogeneity further impedes progress because of the time and expense of developing an animal model for each responsible mutated gene. Recent developments in stem cell biology have the potential to change this balance because patient-derived stem cells provide genetically relevant cellular models that can be used to understand aetiology and as a platform for drug discovery (Mackay-Sim, 2012; Mackay-Sim, 2013).

“Hereditary Spastic Paraplegia” refers to a set of familial disorders that lead to progressive gait disturbances with muscle weakness and spasticity (Salinas et al., 2008). More than 70 genetic loci are associated with HSP signs and symptoms, with autosomal dominant, autosomal recessive or x-linked modes of inheritance, but with some forms “complicated” by additional signs and symptoms such as ataxia, dementia or mental retardation (Novarino et al., 2014; Salinas et al., 2008). We developed a patient-derived stem cell model of HSP using tissue-derived neural stem cells from the olfactory mucosa, the organ of smell in the nose (Abrahamsen et al., 2013). These “olfactory neurosphere-derived” (ONS) cells were derived from patients with mutations in *SPAST*, an “uncomplicated” adult-onset form of HSP, that accounts for over 40% of autosomal-dominant HSP. Several mutations in *SPAST* are represented in this patient cohort; all leading to 50% reduced levels of spastin, the protein encoded by *SPAST*. Spastin is a microtubule severing protein whose activity is exerted preferentially on stable microtubules (Riano et al., 2009). Contrary to expectation, there was a decrease in acetylated α-tubulin, a measure of stabilised microtubules, in human HSP patient-derived stem cells (Abrahamsen et al., 2013). This was accompanied by increased stathmin, a microtubule depolymerising enzyme, and altered intracellular distributions of peroxisomes and mitochondria (Abrahamsen et al., 2013). The intracellular trafficking of peroxisomes was also significantly impaired in HSP patient-derived stem cells (Abrahamsen et al., 2013).

Two tubulin-binding drugs, taxol (paclitaxel) and vinblastine, increased acetylated α-tubulin in patient-derived and control-derived ONS cells in a dose-dependent manner (Abrahamsen et al., 2013). Similarly vinblastine, taxol and nocodazole rescued the pathological phenotype of the spastin-deficient mouse and human cortical neurons *in vitro* (Denton et al., 2014; Fassier et al., 2013). The aim of the present study was to establish doses of tubulin-binding drugs that restore acetylated α-tubulin levels in patient-derived cells to the level in untreated control-derived cells and then to test whether these doses were also effective in restoring peroxisome trafficking to control levels. The hypothesis is that the peroxisome trafficking deficits are caused by the reduced acetylated α-tubulin levels in patient-derived ONS cells. We used a high throughput screening strategy to identify doses of tubulin-binding drugs that restored acetylated α-tubulin using automated image analysis of ONS cells derived from patients with a variety of *SPAST* mutations. Drug doses that restored patient acetylated α-tubulin levels to the level in ONS cells derived from healthy controls were assessed for their ability to increase peroxisome trafficking speeds, using automated analysis of peroxisome movements in living cells. The tubulin-binding drugs tested were taxol, vinblastine, epothilone D and noscapine, which have a variety of tubulin binding sites and effects on microtubule dynamics.

## MATERIALS AND METHODS

### Participants and nasal biopsies

The participants and biopsies are described elsewhere (Abrahamsen et al., 2013). All procedures were carried out in accordance with the human ethics committee of Griffith University and the Northern Sydney and Central Coast Human Research Ethics Committee, and according to guidelines of the National Health and Medical Research Council of Australia.

### Cell culture

Dissociated olfactory tissues were cultured in serum-free medium containing Dulbecco's Modified Minimum Essential Medium (DMEM/F12, Gibco Life Technologies), epidermal growth factor (EGF, Millipore) and basic fibroblast growth factor (FGF2, Millipore) to generate neurospheres from nasal biopsies (Matigian et al., 2010). Free-floating neurospheres were dissociated and grown as adherent cultures (ONS cells) in serum-containing medium, after which they were frozen and stored in liquid nitrogen (Matigian et al., 2010). Frozen aliquots of ONS cells were thawed and cultured for at least 3 days before re-plating for all the experiments described, which were all performed between passages 7–10. All cultures were grown in DMEM/F12 (Gibco) supplemented with 10% fetal bovine serum (FBS) and penicillin/streptomycin (50 units/ml of penicillin and 50 µg/ml of streptomycin, Gibco Life Technologies) at 37°C and 5% CO_2_.

### Effects of microtubule-interrupting drugs on acetylated α-tubulin

ONS cells from patients and controls were grown to 70–80% confluence and re-plated in poly-L-lysine pre-coated 96-well plates (CellCarrier, Perkin Elmer; 3000 cells/well). Then, the cells were cultured for 24 hours in the presence of taxol, vinblastine, epothilone D and noscapone at different concentrations. Cells were then fixed and processed for immunocytochemistry and automated microscopy and high content image analysis, as described below. The drugs were dissolved in DMSO to prepare 5 µM stock solution for taxol, vinblastine, epothilone D and 20 mM stock solution for noscapine. Control solutions contained 0.05% DMSO.

### Immunocytochemistry and cell labelling for acetylated α-tubulin

The cells were fixed in 4% paraformaldehyde dissolved in Hank's balanced salt solution (HBSS, pH 7.4, Gibco Life Technologies) for 15 min at room temperature and permeabilized in 0.1% Triton X-100 in HBSS containing 3% bovine serum albumin (Sigma) for 30 min. Cells were then immunostained with antibodies against acetylated α-tubulin (1:1000 mouse monoclonal; ab24610, Abcam) for 1 hour at room temperature. Cells were washed three times in phosphate-buffered saline (PBS, pH 7.4, Gibco Life Technologies) and incubated for 30 min at room temperature with the secondary antibody (1:400, goat anti-mouse Alexa Fluor 488, A11001, Invitrogen). After two washes with PBS, the cells were stained with CellMask (1:10,000; Life Technologies) for 30 min to stain the cytoplasm and DAPI (1:1000; Life Technologies) for 10 min at room temperature to stain the nucleus. The cells were then washed twice in PBS to remove excess stains.

### Automated microscopy and image analysis of acetylated α-tubulin

For this analysis, cells in 96-well plates were imaged using an automated microscope (Operetta High Content Imaging System, Perkin Elmer). Images were captured at 56 locations per well at 200× magnification at three wavelengths (488 nm, acetylated α-tubulin; 647 nm, CellMask; 350 nm, DAPI). The three images were combined and analyzed using Harmony High Content Analysis Software (Perkin Elmer). The analysis protocol involved the following steps: 1) each cell nucleus was identified using the DAPI stain, 2) the cell cytoplasm was defined from CellMask fluorescence, 3) cells that overlapped the border of the image were excluded from the analysis, 4) acetylated α-tubulin fluorescence intensity was quantified within each cell-cytoplasm region defined as the region between the nucleus (DAPI-labelled) and the outer edge of the cell (CellMask labelled). To reduce experimental variability, all images were taken with the same time and exposure settings and the experiments were designed to include, in the same 96-well plates, cells from different patients and controls and cells exposed to the different drugs and concentrations. Each drug concentration was repeated in triplicate in at least two separate experiments. To obviate observer bias, the image analysis was automated using the same parameters for every image. This analysis provided a mean fluorescence value for acetylated α-tubulin that was the average per-cell value for each patient- and control-derived ONS cell line (n = 9 patients, 8 controls). The patient-control difference in mean per-cell fluorescence is indicative of the patient-control difference in acetylated α-tubulin protein estimated from Western blot analysis in these cells (Abrahamsen et al., 2013).

### Image capture and analysis of the dynamics of peroxisome movement

Cells were cultured on 96-well glass plates (Matriplate; MGB096-1-2-LG-L) coated with poly-L-lysine (10 µg/ml). After 24 h, the cells were transduced for 12 h using a CellLight reagent for peroxisome fluorescence (C10604, CellLight Peroxisome-GFP BacMam 2.0; Life Technologies). This was followed by 24 h exposure to drug treatment. Time-lapse images of fluorescent peroxisomes were then acquired under high magnification (oil immersion objective, 630× total magnification; Zeiss AxioObserver Z1 microscope with AxioCamHs camera). Microscope control and image capture were accomplished using Zeiss AxioVision software (AxioVs40 V 4.8.2.0). In each imaging session, peroxisomes within a field of view were acquired in three dimensions every 2 s for a total recording time of 2 minutes. These movies were then analysed using Imaris software (Imaris×64 6.4.2, Bitplane): 1) the 3D images of each field were collapsed into a single 2D maximum brightness image to allow capture of all peroxisomes in that 3D space; 2) peroxisomes were user-defined based on a filter combining fluorescence intensity and diameter; 3) all the peroxisomes within the field of view were identified automatically using the ‘spot’ function; 4) all spots in the field of view were tracked for the 2 minute observation period using a user-defined dynamic filter to distinguish continuous movement between sequential images; 5) peroxisomes that were not tracked for the full 2 minute observation period were deleted from the analysis; 6) for each peroxisome the mean frame-by-frame speed (µm/s) and total distance travelled during the 2 minute observation period (tracklength, µm) were calculated; 7) the fastest peroxisomes were selected as those in the 90^th^ percentile for mean speed and mean distance travelled during the 2 minute observation period. To reduce experimental variability, all images were taken with the same time and exposure settings and the experiments were designed to include in the same 96-well plates, cells from different patients and controls and cells exposed to the different drugs and concentrations. Each drug concentration was repeated in triplicate in at least two separate experiments. To obviate observer bias, the image analysis was automated using the same parameters for every image. The data are based on analysis of approximately 1000 peroxisomes per cell in 3 cells from each of 6 patient-derived and 6 control-derived ONS cell lines. For the analyses all peroxisomes from all cells from all individuals in each group were pooled. Thus the data present group mean differences between peroxisomes (N = 4818–7554 peroxisomes per group).

### Drug toxicity assay

Drug toxicity was determined using the CytoTox-Glo cytotoxicity assay according to manufacturer-supplied protocol (Promega G9290). Briefly, ONS cells were grown in DMEM/F12 supplemented with 10% FBS until 80% confluence and re-plated in 96 well plates. Cell counts were performed using an automated particle counter (Z1 Coulter Particle Counter, Beckman Coulter); cells were diluted to 2.5×10^4^ cells/ml and 2500 cells (100 µl) were seeded into triplicate wells of 96-well plates and incubated to attach overnight. Then the cells were grown for 24 h in medium containing taxol (0.5 nM), vinblastine (0.5 nM), epothilone D (2 nM) or noscapine (10 µM). The drugs were dissolved in DMSO, which was diluted to 0.05% in these media at the highest drug concentrations. Medium containing same percentage of DMSO was used as negative control and medium containing high concentration of vinblastine (5 µM) as positive control. Further negative controls were duplicates for each drug of drug-only wells without cells in each plate. All plates and reagents were equilibrated to room temperature prior to assay initiation and all results were obtained using a Synergy II plate reader (BioTek). The dead cells in cell populations were determined by measuring “dead-cell protease activity”, which is released from cells that have lost membrane integrity. The total luminescence (total cell number) was acquired through adding 50 µl of lysis reagent/well to lyse all cells. Viability was calculated by comparing the luminescent signal resulting from experimental cell death to total luminescent values.

### Cell proliferation assays

Drug effects on cell proliferation were determined using the CellTiter 96 Aqueous Non-Radioactive Cell proliferation assay (Promega G5421) according to manufacturer-supplied protocol. Briefly, ONS cells were grown in DMEM/F12 supplemented with 10% FBS until 80% confluence and re-plated in 96 well plates. 2500 cells (100 µl) were seeded into triplicate wells of 96-well and allowed to attach overnight. Then the medium was replaced by medium containing taxol (0.5 nM), vinblastine (0.5 nM), epothilone D (2 nM) or noscapine (10 µM) and cells were kept in 37°C and 5% CO_2_ for 24 h or 7 d (the medium was refreshed every 3 days). The drugs were dissolved in DMSO, which was diluted to 0.05% in these media. Medium containing the same percentage of DMSO was used as negative control and medium containing high concentration of vinblastine (5 µM) as positive control. Further negative controls were duplicates for each drug of drug-only wells without cells in each plate. Two hours before measurement, 20 µl of combined 3-(4,5-dimethylthiazol-2-yl)-5-(3-carboxymethoxyphenyl)-2-(4-sulfophenyl)-2H-tetrazolium (MTS)/phenazine methosulfate (PMS) solution was added into each well of the 96 well assay plate containing 100 µl of culture medium. The absorbance at 490 nm was recorded using a Synergy II plate reader (BioTek). Data were normalized to DNA content, determined using supplied protocols (CyQUANT-NF, Invitrogen; 50 µl prepared reagent per well, including medium-only control wells). For each cell line, triplicate values from each assay were averaged and a signal:DNA content ratio determined.

Drug effects on cell proliferation were also determined by flow cytometry after labelling cells during DNA synthesis with the thymidine analog, EdU. Briefly, ONS cells were grown in DMEM/F12 supplemented with 10% FBS until 80% confluence and 0.5×10^6^ cells were re-plated in T25 flasks in the same medium overnight to allow the cells to attach. Then the medium was replaced by medium containing taxol (0.5 nM), vinblastine (0.5 nM), epothilone D (2 nM) or noscapine (10 µM) and cells were kept in 37°C and 5% CO_2_ for 48 h, in the presence of EdU (10 µM). Cells of each cell line cultured in medium containing 0.05% DMSO were used as untreated controls and cells cultured in medium without EdU were used as negative controls. The cells were harvested and fixed in 4% paraformaldehyde in HBBS, pH 7.4 for 10 minutes at room temperature. EdU incorporation was identified using a Click-iT EdU Flow Cytometry Assay Kit (Invitrogen) according to the manufacturer's instruction. The percentage of EdU positive cells was analyzed by flow cytometry (BD FACSAria flow cytometer). At least 10,000 cells were analysed for each sample. Analysis was performed using FloJo version 7.2.5 (Tree Star, Ashland, OR).

### Protein analysis

ONS cells were cultured in T25 flasks in DMEM/F12 supplemented with 10% FBS until 70% confluence and treated for 24 hours [DMSO (0.05%); taxol (0.5 nM), vinblastine (0.5 nM), epothilone D (2 nM) or noscapine (10 µM)]. Cells were washed twice in PBS and lysed with 100 µl RIPA (25 mM Tris-HCl pH 7.4, 2 mM EDTA, 150 mM NaCl, 1% Triton X-100, 1 mM NaF, 0.25% sodium deoxycholate, 0.2 mM Na_3_VO_4_, 1× protease inhibitors cocktail; Roche). The protein concentration of cell lysates was determined using a DC protein assay kit (Bio-Rad). Equal total amount of proteins (10 µg) were loaded onto a 10% denaturing sodium dodecyl sulfate-polyacrylamide gel for electrophoresis. After electrophoresis, the gels were electroblotted onto polyvinylidine difluoride membranes, blocked with Tris-buffered saline containing 5% milk and 0.1% Tween 20 for 1 hour and incubated overnight at 4°C with the primary antibody [anti-acetylated α-tubulin, 1:1000, ab24610, Abcam; anti-PEX14, 1:1000, (Nguyen et al., 2006); anti-GAPDH, 1:2500, Covance]. Immunoreactivity was visualized with a secondary antibody (horseradish peroxidase (HRP)-conjugated anti-rabbit or HRP-conjugated anti-mouse; 1:5000), using the enhanced chemiluminescence system according to the manufacturer's instructions (Immobilon western chemiluminescent HRP substrate; Millipore). The quantification of immunoblotting was analyzed using the Quantity One program (Bio-Rad), normalizing against GAPDH immunoreactivity in each sample as an internal control.

### Statistical analysis

Data are expressed as mean (± SEM). Data were compared with one-way Analysis of Variance (ANOVA) with Dunnett's post-hoc multiple comparisons tests using GraphPad (v6, Prism). Significance was assumed with a criterion of α<0.05.

## RESULTS

The patient-derived cells (n = 9) and control-derived cells (n = 8) used here are described elsewhere (Abrahamsen et al., 2013). The patients were all diagnosed with adult onset Hereditary Spastic Paraplegia by a neurologist (CS) and in each case a mutation in *SPAST* was identified: the mutations included single nucleotide substitutions, insertions and deletions (Abrahamsen et al., 2013).

### Low dose tubulin-binding drugs restored acetylated α-tubulin levels in patient-derived cells

**Fig. 1.**
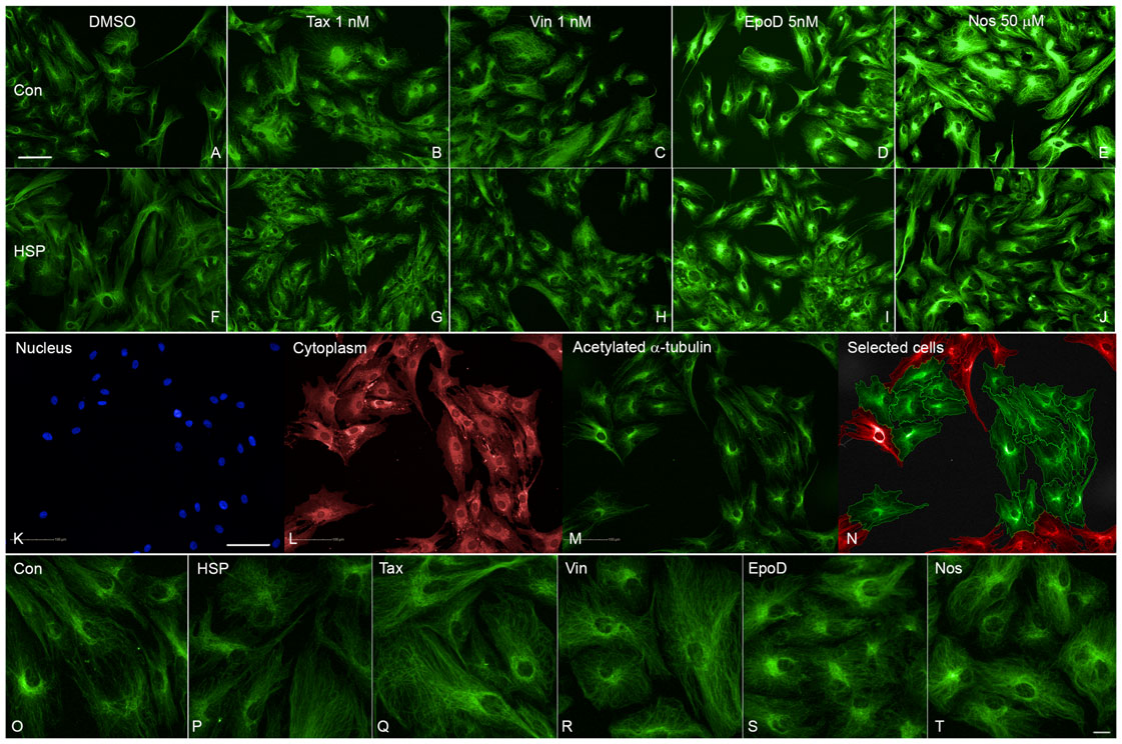
Tubulin-binding drugs increased acetylated α-tubulin levels in patient-derived cells. (A–E) Images of a control-derived ONS cell line showing increased fluorescence and disrupted acetylated α-tubulin microtubules after exposure to DMSO diluent alone and to moderate doses of taxol (Tax), vinblastine (Vin), epothilone D (EpoD) and noscapine (Nos) at the concentrations indicated. (F–J) Images of a patient-derived ONS cell line showing similar effects of moderate doses of taxol (Tax), vinblastine (Vin), epothilone D (EpoD) and noscapine (Nos) at the concentrations indicated above. (K–N) Images of the same field of cells showing (K) nuclei (blue, DAPI), (L) cell cytoplasm (red, CellMask), and (M) acetylated α-tubulin (green, immunofluorescence). (N) Image showing cells included for automated analysis (green) or excluded (red). Automated identification of cell boundaries is shown as lines. acetylated α-tubulin immunofluorescence (green) was quantified within the cell boundary and the nuclear boundary. (O–T) Higher power images showing acetylated α-tubulin immunoreactivity in (O) control-derived ONS cells (Con) and (P) patient-derived ONS cells (HSP) exposed to DMSO only and the same patient-derived cell line to low doses of taxol (0.5 nM, Tax), vinblastine (0.05 nM, Vin), epothilone D (2 nM, EpoD) and noscapine (10 µM, Nos). Scale bars: 100 µm (A–N), 20 µm (O–T).

We previously showed that patient-derived ONS cells had a 50% reduction of acetylated α-tubulin, compared to control-derived ONS cells and the tubulin-binding drugs, taxol and vinblastine, increased acetylated α-tubulin in patient-derived and control-derived cells in a dose-dependent manner (Abrahamsen et al., 2013). These drugs have different tubulin-binding mechanisms, so we tested two further compounds that have other tubulin-binding mechanisms, epothilone D and noscapine. After pilot experiments to establish appropriate dose ranges, the cells were cultured in 96 well plates for 24 h in the presence of epothilone D (0.5 nM, 1 nM, 5 nM) or noscapine (1 µM, 5 µM, 10 µM, 50 µM). All drugs at moderate doses increased acetylated α-tubulin immunofluorescence levels in both control-derived and patient-derived cells (Fig. 1A–J). Epothilone D and noscapine increased cellular acetylated α-tubulin in a dose-dependent manner in these low dose ranges similar to increases induced by taxol and vinblastine (supplementary material Fig. S1) (Abrahamsen et al., 2013). From the dose response curves, a concentration of each drug was chosen that raised the acetylated α-tubulin in HSP patient-derived cells to the level in control-derived cells (supplementary material Fig. S1). These selected concentrations (0.5 nM taxol, 0.5 nM vinblastine, 2 nM epothilone D, 10 µM noscapine) were subsequently used for all subsequent experiments.

**Fig. 2.**
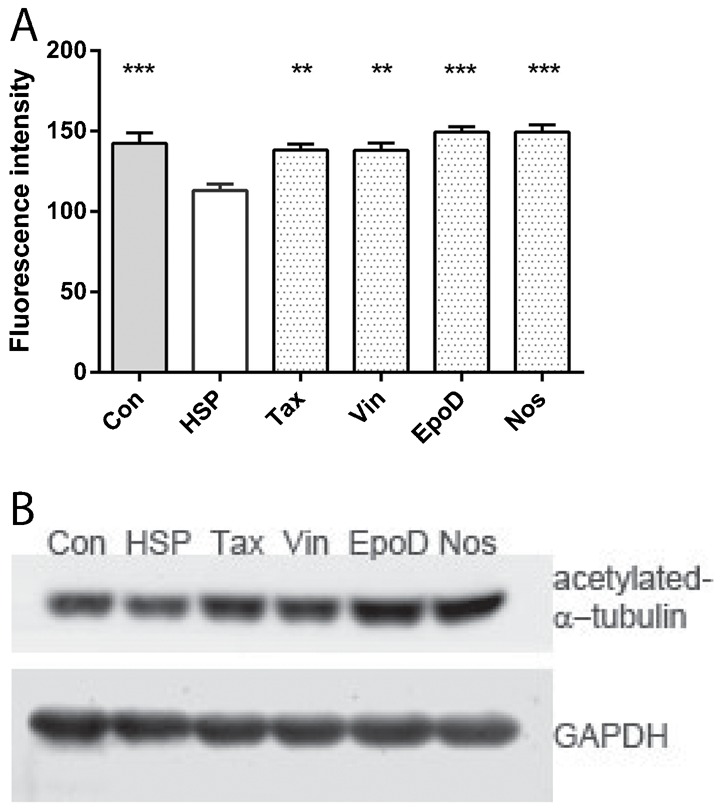
Tubulin-binding drugs rescued acetylated α-tubulin levels in patient-derived cells. (A) acetylated α-tubulin fluorescence intensity was significantly different among the groups (ANOVA, p<0.0001). Data are mean ± SEM of the mean per cell fluorescence intensity averaged across cell lines: control-derived cells, n = 8 cell lines from 8 different individuals; patient-derived cells, n = 9 cell lines from 9 different individuals. Con: control-derived cell lines treated with DMSO alone; HSP: patient-derived cell lines treated with DMSO alone; Tax: patient-derived cell lines treated with 0.5 nM taxol; Vin: patient-derived cell lines treated with 0.5 nM vinblastine; EpoD: patient-derived cell lines treated with 2 nM epothilone D; Nos: patient-derived cell lines treated with 10 µM noscapine. Post-hoc tests indicated that the means of all other groups were significantly different from the HSP group: *p<0.05; **p<0.01; ***p<0.001. (B) Western blot showing acetylated α-tubulin levels compared to GAPDH control levels in cell lines treated with the same drugs.

High content screening with quantitative image analysis was used to measure acetylated α-tubulin levels in drug-treated patient-derived cells in comparison to untreated patient-derived and control-derived cells exposed to 0.05% DMSO, the concentration used to dissolve the drugs. The automated analysis identified the nuclei (DAPI labelled, Fig. 1K), the cell cytoplasm (CellMask labelled, Fig. 1L) and acetylated α-tubulin immunoreactivity (Fig. 1M). The nucleus and cytoplasm were used to generate the region of interest for each cell: the cytoplasm between the nucleus and the cell boundary and cells not fully contained within the field of view were excluded from the analysis (Fig. 1N). At the selected doses the drugs had subtle effects on the acetylated α-tubulin levels in patient-derived cells that were not very obvious visually (Fig. 1O–T) but quantification showed that all drugs restored the acetylated α-tubulin levels in patient-derived cells to levels in untreated control-derived cells (Fig. 2A). ANOVA indicated a significant effect of treatment (F5,47 = 9.032; p<0.0001). Dunnett's post-hoc multiple comparisons indicate that acetylated α-tubulin levels in untreated patient-derived cells were significantly different from untreated controls (p<0.001) and patient-derived cells treated with taxol (p<0.01), vinblastine (p<0.01), epothilone D (p<0.001) and noscapine (p<0.001). The subtle effects of the drugs were confirmed by Western blotting (Fig. 2B).

### Low dose tubulin-binding drugs restored peroxisome trafficking in patient-derived cells

**Fig. 3.**
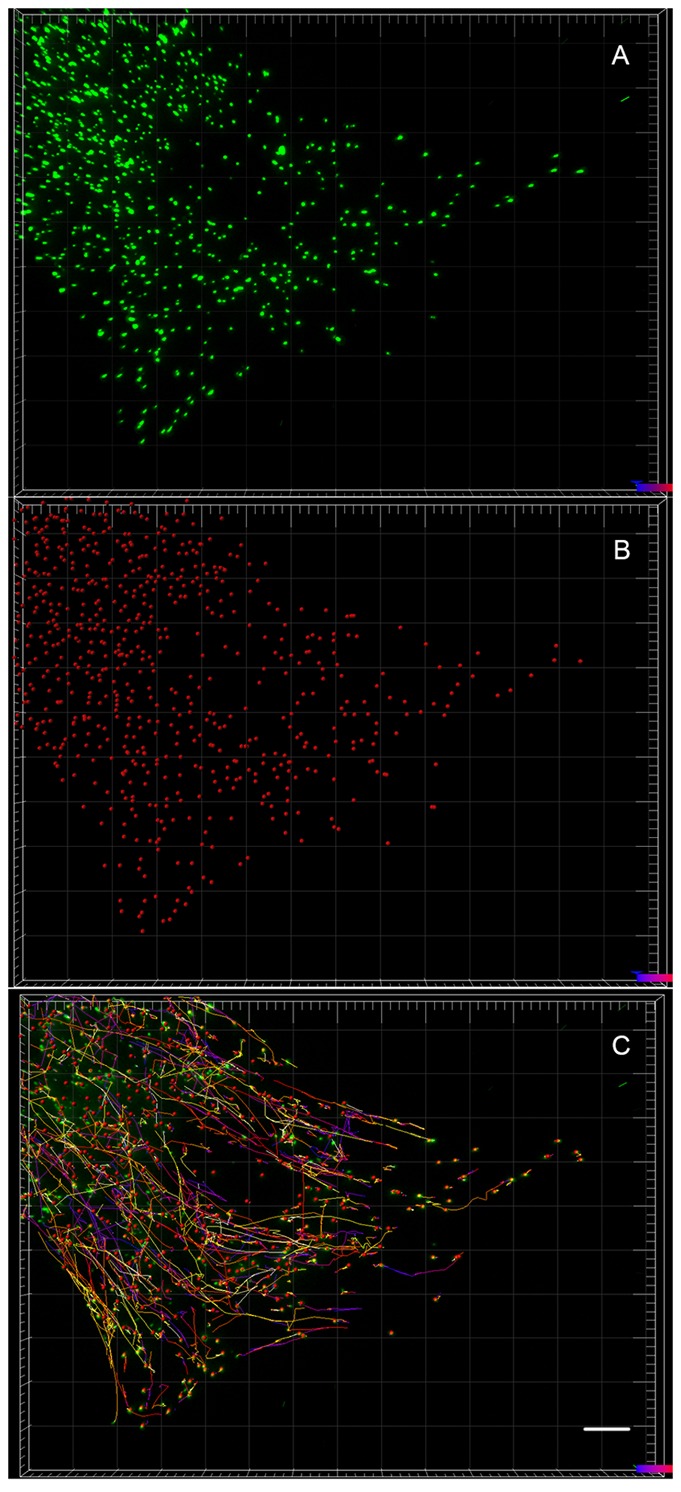
Automated tracking of peroxisomes. (A) Images of the labelled peroxisomes in a cell. (B) The same cell showing the peroxisomes represented as dots after automated identification. (C) The same cell showing the paths taken by all the peroxisomes at the end of the 2 minute observation period. Scale bar: 10 µm.

**Fig. 4.**
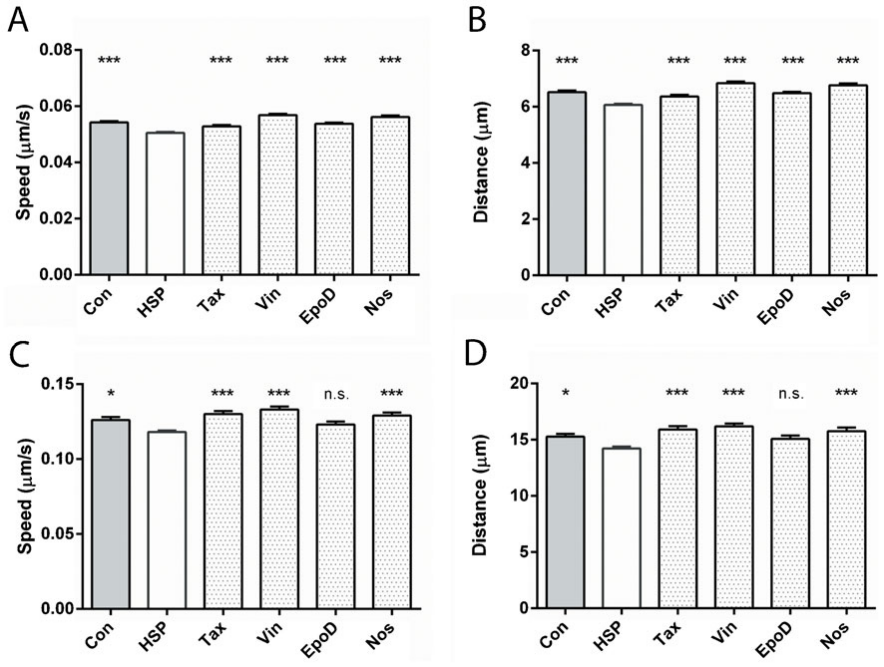
Tubulin-binding drugs rescued peroxisome trafficking in patient-derived cells. (A) Average speed of all peroxisomes (ANOVA, p<0.001). (B) Average distance travelled by all peroxisomes (ANOVA, p<0.01). (C) Average speed of peroxisomes undergoing tubulin-based (saltatory) movement, (ANOVA, p<0.0001). (D) Average distance travelled of peroxisomes undergoing tubulin-based (saltatory) movement (ANOVA, p<0.0001). Data are mean ± SEM. Con: untreated control-derived cell lines; A,B: n = 5935, 5935; C,D: n = 620, 614. HSP: untreated patient-derived cell lines; A,B: n = 7554, 7554; C,D: n = 613, 608. Tax: patient-derived cell lines treated with 0.5 nM taxol; A,B: n = 5871, 5871; C,D: n = 554, 553. Vin: patient-derived cell lines treated with 0.5 nM vinblastine; A,B: n = 6123, 6123; C,D: n = 705, 697. EpoD: patient-derived cell lines treated with 2 nM epothilone D; A,B: n = 6725, 6721; C,D: n = 653, 648. Nos: patient-derived cell lines treated with 10 µM noscapine; A,B: n = 4818, 4818; C,D: n = 574, 520. Post-hoc tests indicated that most means of all other groups were significantly different from the HSP group: n.s., >0.05; *p<0.05; ***p<0.001.

Peroxisome movement was quantified by automated identification and selection of all peroxisomes in a cell and tracking their movement for 2 minutes (Fig. 3). Peroxisomes in control and patient-derived cells exhibited Brownian and saltatory movements (supplementary material Movies 1 and 2), with a speeds ranging from 0–0.6 µm/s. Peroxisomes involved in saltatory movement are obvious visually (supplementary material Movies 1 and 2). Peroxisomes travelled more slowly, on average, in untreated patient-derived cells compared to control-derived cells with the mean peroxisome speed in patient-derived cells 93% of control-derived cells. All the drugs increased mean peroxisome speeds to close to the levels in untreated control-derived cells (Fig. 4A). ANOVA indicated a significant effect of treatment (F5,37020 = 30.00; p<0.0001). Dunnett's post-hoc multiple comparisons indicate that mean peroxisome speed in untreated patient-derived cells was significantly different from untreated controls (p<0.0001) and after treatment with taxol (p<0.001), vinblastine (p<0.0001), epothilone D (p<0.0001) and noscapine (p<0.0001). As expected from these mean speeds, peroxisomes travelled shorter distances in untreated patient-derived cells compared to control-derived cells with the mean peroxisome distance travelled in patient-derived cells 93% of control-derived cells. All the drugs increased the mean peroxisome distances travelled in patient-derived cells to close to the levels in untreated control-derived cells (Fig. 4B). ANOVA indicated a significant effect of treatment (F5,37020 = 28.99; p<0.0001). Dunnett's post-hoc multiple comparisons indicate that mean peroxisome distance in untreated patient-derived cells was significantly different from untreated controls (p<0.0001) and after treatment with taxol (p<0.001), vinblastine (p<0.0001), epothilone D (p<0.0001) and noscapine (p<0.0001).

Mean peroxisome speeds and distances travelled include the data from all peroxisomes in the cell, most of which slowly vibrate and do not show fast movement, the latter requiring microtubules (Rapp et al., 1996; Wiemer et al., 1997). To get an indication of the drug effects on the saltatory movement of peroxisomes we selected the fastest peroxisomes, those with in the 90^th^ percentile in mean speed (Fig. 4C). Again, the fastest peroxisomes in the patient-derived cells had a mean speed of 93% of control-derived cells. ANOVA indicated a significant effect of treatment (F5,3713 = 8.448; p<0.0001). Dunnett's post-hoc multiple comparisons indicate that mean peroxisome distance in untreated patient-derived cells was significantly different from untreated controls (p<0.05) and after treatment with taxol (p<0.001), vinblastine (p<0.0001) and noscapine (p<0.001). As another indicator of saltatory movement we selected the peroxisomes in the 90^th^ percentile in distance travelled. Again, the fastest peroxisomes in the patient-derived cells had a mean distance travelled of 93% of control-derived cells. ANOVA indicated a significant effect of treatment (F5,3634 = 6.905; p<0.0001). Dunnett's post-hoc multiple comparisons indicate that mean peroxisome distance in untreated patient-derived cells was significantly different from untreated controls (p<0.05) and after treatment with taxol (p<0.001), vinblastine (p<0.0001) and noscapine (p<0.001). Epothilone D led to a small increase in the mean speed and distance travelled of the fastest peroxisomes compared to untreated cells, but the effect was not statistically significant.

### Low-dose tubulin-binding drugs were not toxic to patient-derived cells

**Fig. 5.**
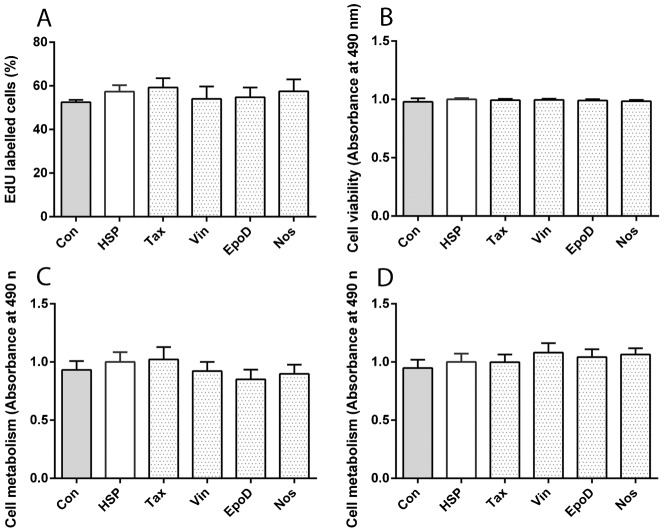
Tubulin-binding drugs were not toxic. (A) Cell proliferation was not different among the groups, assessed from the percentage of cells labelled with EdU. (B) Cell viability was not different among the groups, assessed with the CytoTox assay. (C) Cell metabolism was not different among the groups at after 1 d drug exposure, assessed with the MTS assay. (D) Cell metabolism was not different among the groups at after 7 d drug exposure, assessed with the MTS assay. Data are mean ± SEM. Con: untreated control-derived cell lines, n = 8; HSP: untreated patient-derived cell lines, n = 8; Tax: patient-derived cell lines treated with 0.5 nM taxol, n = 8; Vin: patient-derived cell lines treated with 0.5 nM vinblastine, n = 8; EpoD: patient-derived cell lines treated with 2 nM epothilone D, n = 8; Nos: patient-derived cell lines treated with 10 µM noscapine, n = 8.

Tubulin-binding drugs at clinically relevant doses are designed for their toxicity against proliferating cancer cells. Accordingly the drugs were tested in an array of cell proliferation, toxicity and metabolic assays, none of which demonstrated any effects of the drugs (Fig. 5). Cell proliferation was assessed by quantifying the proportion of EdU-positive cells in patient-derived and control-derived cells after 3 d (Fig. 5A). ANOVA indicated that there was no significant effect of treatment (F5,22 = 0.2743, p = 0.9223). Cell toxicity was assessed using the Cytotox assay that quantifies dead-cell protease activity in patient-derived and control-derived cells after 1 d (Fig. 5B). ANOVA indicated that there was no significant effect of treatment (F5,42 = 0.2370, p = 0.9440). Cell metabolism was assessed using MTS metabolism assays in patient-derived and control-derived cells after 1 d and 7 d (Fig. 5C,D). ANOVA indicated that there was no significant effect of treatment after 1 d (F5,42 = 0.5674, p = 0.7244) or 7 d (F5,42 = 0.5106, p = 0.7666).

**Fig. 6.**
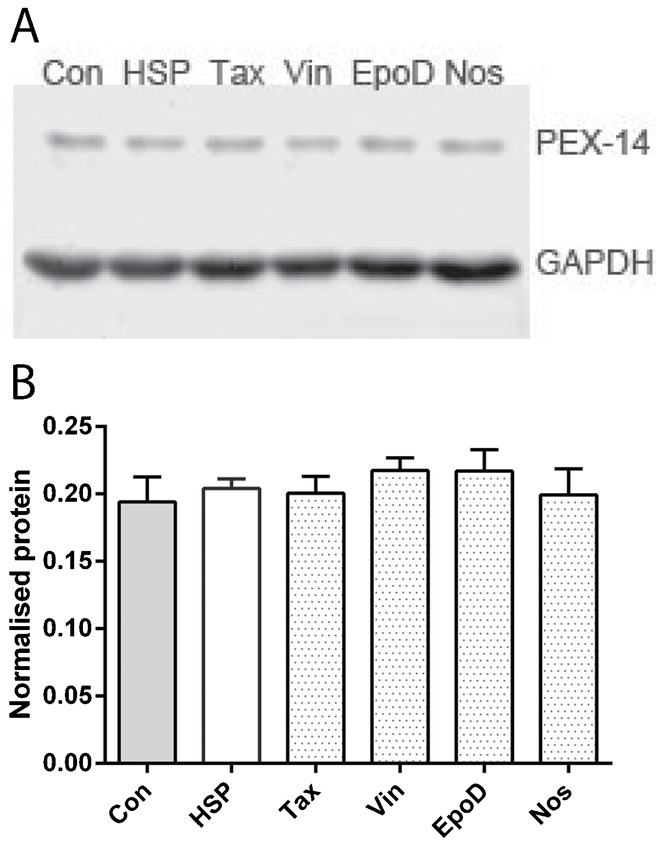
Tubulin-binding drugs did not affect PEX14 levels. (A) Western blot showing PEX14 expression in the different groups, with GAPDH, the loading control. (B) Semi-quantitative analysis of PEX14 levels among the groups showing the average ratio of PEX14 normalised to GAPDH, the loading control. Data are mean ± SEM. Con: untreated control-derived cell lines, n = 3; HSP: untreated patient-derived cell lines, n = 3; Tax: patient-derived cell lines treated with 0.5 nM taxol, n = 3; Vin: patient-derived cell lines treated with 0.5 nM vinblastine, n = 3; EpoD: patient-derived cell lines treated with 2 nM epothilone D, n = 3; Nos: patient-derived cell lines treated with 10 µM noscapine, n = 3.

The cytoskeleton plays an important role in peroxisome biogenesis; altered cytoskeleton dynamics may affect peroxisome biogenesis (Nguyen et al., 2006). To exclude the possible impacts of peroxisome biogenesis on assessments of peroxisome movement, we evaluated the effects of drug treatments on the levels of a peroxisomal membrane protein (PEX14) as a measure of peroxisome abundance. Western blot analysis showed no difference in PEX14 levels before and after the drug treatments (Fig. 6A,B).

## DISCUSSION

In this study functional deficits in HSP patient-derived stem cell biology were used as an assay for drug discovery. We identified low concentrations of four tubulin-binding drugs that increased the acetylated α-tubulin levels in patient-derived stem cells to the levels in control-derived stem cells. Next, we showed that these concentrations increased peroxisome trafficking speeds and distance travelled in patient-derived stem cells to the levels in control-derived stem cells. Then, we showed that at these concentrations, the drugs were not cytotoxic and none affected cell proliferation. At a practical level we demonstrate a methodology for drug discovery using these cells, based on high content screening for a histological cell phenotype (acetylated α-tubulin) subsequently validated for a cell function (intracellular trafficking). This study thus presents some initial therapeutic drug candidates for further development.

The difference between patient-derived cells and control-derived cells in acetylated α-tubulin immunofluorescence levels was 73%. Immunofluorescence is not linear and this difference may be an underestimate as we previously observed a 50% reduction in acetylated α-tubulin measured by semi-quantitative Western analysis (Abrahamsen et al., 2013). The differences in peroxisome speed and distance travelled were 93%, both for estimates based on all peroxisomes and for those in the fastest 10%. Functionally, this small difference may not be trivial especially in long axons such as those in the corticospinal tract. Given the data here, for example, the fastest peroxisomes would take 2.2 hr to travel down a 1 m long control-derived axon and 2.35 hr to travel down a patient-derived axon of the same length. Peroxisomal catalase is essential for synaptic regulation of reactive oxygen species and slowing of peroxisome trafficking could compromise synaptic function and axonal health (Massaad and Klann, 2011). This could be exacerbated with age as oxidative damage accumulates and anti-oxidative defences decline (Massaad and Klann, 2011), potentially leading to the adult onset that is associated with HSP associated with *SPAST* mutations (Salinas et al., 2008).

### Tubulin acetylation and peroxisome trafficking

In our previous study we demonstrated reduced levels of acetylated tubulin in HSP patient cells (Abrahamsen et al., 2013). Acetylation is a marker for stabilised microtubules (Piperno et al., 1987). Stable, long-lived microtubules are also detyrosinated (Janke and Kneussel, 2010) and in neurons acetylated and detyrosinated microtubules are enriched in proximal axons (Baas et al., 1991). Post-translation modifications of microtubules by acetylation, detyrosination and polyglutamination may all contribute to the targeting of molecular motors and their trafficking cargos to specific microtubule tracks (Janke and Kneussel, 2010). This may be especially critical in neurons because of the different patterns of post-translational modifications in axons, soma and dendrites (Janke and Kneussel, 2010). For example, kinesin-5 binding to α-tubulin is acetylation dependent (Bulinski, 2007; Reed et al., 2006) whereas KIF17 and KIF1A do not show selectivity for stable microtubules (Cai et al., 2009). Microtubule acetylation speeds anterograde traffic flow (Bulinski, 2007) and HDAC6 inhibition enhances mitochondrial movement in hippocampal neurons through increasing the level of acetylated tubulin (Chen et al., 2010). HSP patient ONS cells have less acetylated α-tubulin and altered intracellular distributions of mitochondria and peroxisomes and slower peroxisome trafficking speeds, compared to control ONS cells (Abrahamsen et al., 2013). Our hypothesis is that the impaired peroxisome movement in patient cells is due to the down-regulation of acetylated α-tubulin in HSP cells. We show here that by raising acetylated α-tubulin levels in the patient-derived cells, peroxisome trafficking speed is restored to levels in control-derived cells.

The mechanism, by which low concentrations of tubulin-binding drugs affect peroxisome trafficking, is not clear. It is possible that drug treatments alter peroxisome movement by mechanisms other than increasing microtubule stabilisation. For instance, increased peroxisome speeds may result from the reintegration of the microtubule lattice after drug treatment, in addition to the increased acetylated α-tubulin (Kan et al., 2011). A common feature of tubulin-binding compounds is a linkage to assembly, either the stabilization of a microtubule lattice by compounds like taxol or epothilone A, or the preferential formation of alternate lattice contacts and polymers at microtubule ends by compounds like colchicine (Amos, 2011). It is interesting that we found similar effects on stabilised microtubules at these very low doses of drugs with different binding sites and mechanisms of microtubule disruption. Taxol and epothilones bind to the same site on β-tubulin and promote assembly of microtubules, to stabilise the microtubule lattice, increase microtubule polymerisation and promote microtubule bundling (Amos, 2011; Bollag et al., 1995; Checchi et al., 2003). Vinblastine inhibits tubulin assembly into microtubules (Gigant et al., 2005). Noscapine alters the steady-state dynamics of microtubule assembly by increasing the time microtubules spend in a paused state during periods of assembly (Zhou et al., 2003).

### Organelle trafficking in Hereditary Spastic Paraplegia

Axon loss is a feature of the pathology of patients with HSP, evident as reductions in the corticospinal motor tract as well as the sensory tracts in the upper spinal cord (Deluca et al., 2004). Reductions in corticospinal tract integrity are also seen in living patients with *SPAST* and *SPG7* mutations (Duning et al., 2010). At the cellular level, axonal pathology was evident in the corticospinal tract of patients with *SPAST* mutations demonstrated by swellings of axons that were strongly immunoreactive for amyloid precursor protein, a marker for membrane bound organelles that are cargoes of fast axonal transport (Kasher et al., 2009). Further verification that axonal swellings may be a unifying pathology is given by evidence that cortical neurons differentiated from induced pluripotent stem cells from a *SPAST* HSP patient also have increased occurrences of axonal swellings (Denton et al., 2014).

SPG4 is the mouse ortholog of the human *SPAST* gene encoding for spastin. Mice homozygous for SPG4 mutations exhibit many of the pathologies of humans heterozygous for *SPAST* mutations, including degeneration of axons in ascending and descending tracts of the spinal cord (Kasher et al., 2009; Tarrade et al., 2006) and axonal swellings in corticospinal tract axons present at 12 months but not at 4 months, nor in control mice (Tarrade et al., 2006). The axonal swellings contained a large number of organelles, including mitochondria, lysosomes and peroxisomes, disorganised neurofilaments, and in older animals there was a depletion of microtubules (Tarrade et al., 2006). Cortical neurons cultured from mutant mice also developed focal swellings in the distal sections of their neurites (Kasher et al., 2009; Tarrade et al., 2006).

*SPG4* mutant mouse cortical neurons *in vitro* were able to transport the cholera b subunit through the axonal swellings to the cell soma even though the protein accumulated in the swellings, demonstrating that retrograde transport was stalled but not abolished (Fassier et al., 2013). Similarly, the anterograde transport of amyloid precursor protein-containing vesicles and mitochondria was reduced but not blocked in *SPG4* mutant mice (Kasher et al., 2009), consistent with microtubule-dependent cargo trafficking stalling in the axonal swellings but then resuming, leading to a progressive accumulation of organelles within the swelling. Retrograde transport of mitochondria was impaired in cortical neurons generated from induced pluripotent stem cells derived from a *SPAST* HSP patient (Denton et al., 2014). Although there may be cell-type, individual, or species differences it is clear from all studies that *SPAST/SPG4* mutations lead to deficits in axonal trafficking of several organelles. Our experiments confirm this cellular pathology through demonstrating significant slowing of peroxisome trafficking speed in *SPAST*-HSP patient derived ONS cells (Abrahamsen et al., 2013). This was evidently not dependent on morphological abnormality, such as axonal swellings. That it was corrected by low doses of tubulin-binding drugs suggests that the primary mechanism for the trafficking deficit is a deficit in the stabilised microtubule network. Tubulin-binding drugs also reduced the proportion of *SPG4* mutant mouse cortical neurons with axonal swellings (Fassier et al., 2013) and in human cortical neurons derived from *SPAST* patient (Denton et al., 2014).

It is encouraging that data from mutant mouse and human patient-derived cells are showing deficits in the same domains of cell biology giving hope that aetiology and drug discovery based on the animal model will translate directly to human. A puzzle remains concerning the effect of *SPAST* mutations on the cellular level of acetylated α-tubulin because it is reduced in patient-derived ONS cells (Abrahamsen et al., 2013; present study) but elevated in patient iPS-derived cortical neurons (Denton et al., 2014). In both models vinblastine ameliorated disease-associated deficits [peroxisome trafficking in ONS cells, (Abrahamsen et al., 2013); axonal swellings in iPS-derived neurons (Denton et al., 2014)].

### Tubulin-binding drugs as potential therapies for Hereditary Spastic Paraplegia

Each of the tubulin drugs applied here is used clinically, or has passed Phase I clinical trials, making them candidates for “re-purposing” from cancer therapies. The doses effective for blocking mitosis and causing apoptosis of cancer cells is normally 100–1000 fold higher than those used in this study (Axiak-Bechtel et al., 2013). All are tolerable for short periods as anti-cancer therapies, although peripheral neuropathy is among the side effects of vinblastine-related treatment for cancer (Gidding et al., 1999). The low doses used here were non-toxic to HSP patient-derived ONS cells and did not affect cell proliferation or viability even after 7 d *in vitro*. For clinical use in HSP a drug may have to be taken on a chronic basis and the safety profile for even very low drug doses would have to be confirmed.

Neither taxol nor vinblastine can penetrate the blood–brain barrier and would not be available to affect microtubule stability in corticospinal motor neurons. Epothilone D is more water soluble than taxol and vinblastine (Altmann et al., 2000). It readily crosses the blood–brain barrier and is cleared more slowly from brain than plasma (Brunden et al., 2010). Epothilone D is already in early phase clinical trials for Alzheimer's disease, based on its ability to improve microtubule density, axonal density and cognition in a mouse model, with no dose-limiting side effects (Brunden et al., 2011; Zhang et al., 2012). Noscapine is water soluble, non-toxic and is safe enough to be given orally as an anti-tussive (Aneja et al., 2007). It penetrates the blood–brain barrier and is active against brain tumours (Jhaveri et al., 2011). This ability to enter the brain makes these drugs attractive candidates for further development.

### Conclusions

In summary, the strategy used here has 1) identified disease-associated reductions in acetylated α-tubulin content and peroxisome trafficking (Abrahamsen et al., 2013); 2) identified concentrations of tubulin-binding drugs that restored acetylated α-tubulin content in patient-derived cells; and 3) demonstrated that those drug concentrations restored acetylated α-tubulin and the peroxisome trafficking deficit in HSP patient-derived cells. This approach could be applied in a high throughput screening strategy to identify other drug candidates that improve intracellular trafficking in Hereditary Spastic Paraplegia. The strategy has several advantages for high throughput screening. First, the patient-derived ONS cells are relatively easily obtained and relatively simple to grow and maintain. Second, ONS cells can be banked and grown homogeneously in quantities necessary for screening. Third, hits from an initial high throughput assay (image analysis of acetylated α-tubulin content in fixed cells) were validated in a functional assay (live imaging and quantification of peroxisome movement). We have focussed here on drug candidates based on approved drugs. Re-purposing of approved drugs could reduce considerably the costs for bringing a drug to market, a crucial aspect when considering rare neurological disorders.

## Supplementary Material

Supplementary Material
