# Supplementary material for: Low dose tubulin-binding drugs rescue peroxisome trafficking deficit in patient-derived stem cells in Hereditary Spastic Paraplegia
Source: Biol Open. 2014 May 23;3(6):494–502. doi: 10.1242/bio.20147641 (PMC4058084; doi:10.1242/bio.20147641)
Supplement: Supplementary Material [file supp_3_6_494__index.html]

Low dose tubulin-binding drugs rescue peroxisome trafficking deficit in patient-derived stem cells in Hereditary Spastic Paraplegia — Supplementary Material 

# Low dose tubulin-binding drugs rescue peroxisome trafficking deficit in patient-derived stem cells in Hereditary Spastic Paraplegia

## bio.20147641 Supplementary Material

**Files in this Data Supplement:**

- Supplementary Material - Yongjun Fan et al. doi: 10.1242/bio.20147641
- Movie 1 - **Movie 1. Representative time-lapse movie showing peroxisomes in a control-derived ONS cell.** Example of peroxisomes moving within cells. Compressing the images into this movie format severely reduces image quality. Peroxisomes are green dots. Most show vibratory, Brownian-like motion while some show bursts of rapid, saltatory microtubule-based movement. The movie was captured for duration of 2 minutes at 2 second intervals.
- Movie 2 - **Movie 2. Representative time-lapse movie showing peroxisomes in a HSP patient-derived ONS cell.** Example of peroxisomes moving within cells. Compressing the images into this movie format severely reduces image quality. Peroxisomes are green dots. Most show vibratory, Brownian-like motion while some show bursts of rapid, saltatory microtubule-based movement. The movie was captured for duration of 2 minutes at 2 second intervals.
