# Supplementary material for: Low dose tubulin-binding drugs rescue peroxisome trafficking deficit in patient-derived stem cells in Hereditary Spastic Paraplegia
Source: Biol Open. 2014 May 23;3(6):494–502. doi: 10.1242/bio.20147641 (PMC4058084; doi:10.1242/bio.20147641)
Supplement: Supplementary Material [file supp_bio.20147641_bio.20147641-s1.pdf]

Supplementary Material
Yongjun Fan et al. doi: 10.1242/bio.20147641

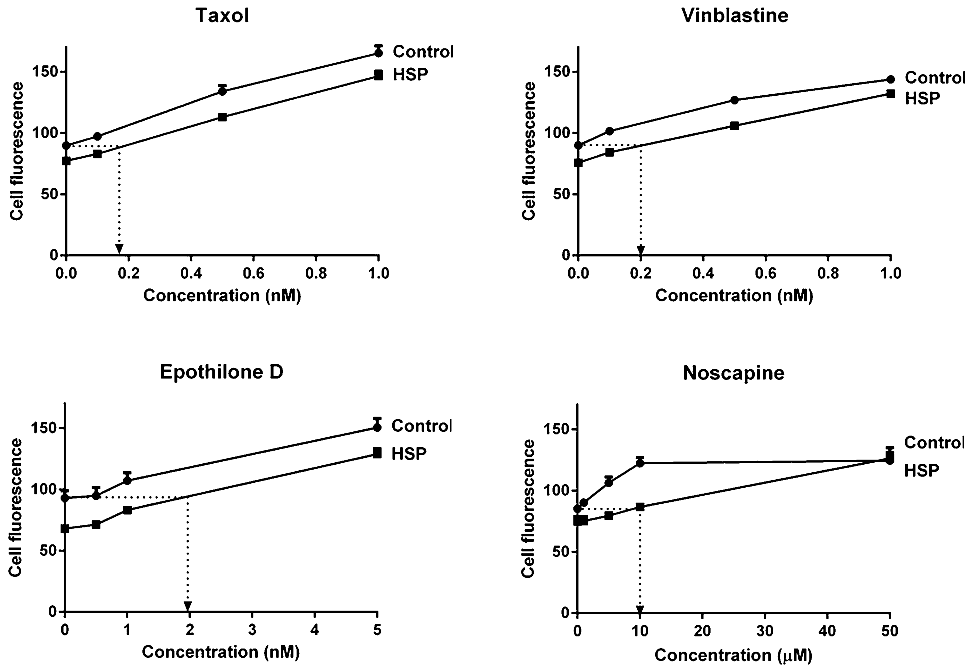

Fig. S1. All tubulin-binding drugs increased acetylated α-tubulin in patient-derived and control-derived cells. Drug doses are indicated that increased acetylated α-tubulin in patient-derived cell lines to untreated control-derived cell levels (dashed lines).

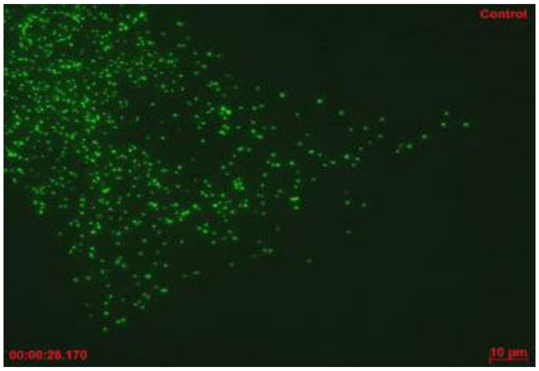

Movie 1. Representative time-lapse movie showing peroxisomes in a control-derived ONS cell. Example of peroxisomes moving within cells. Compressing the images into this movie format severely reduces image quality. Peroxisomes are green dots. Most show vibratory, Brownian-like motion while some show bursts of rapid, saltatory microtubule-based movement. The movie was captured for duration of 2 minutes at 2 second intervals.

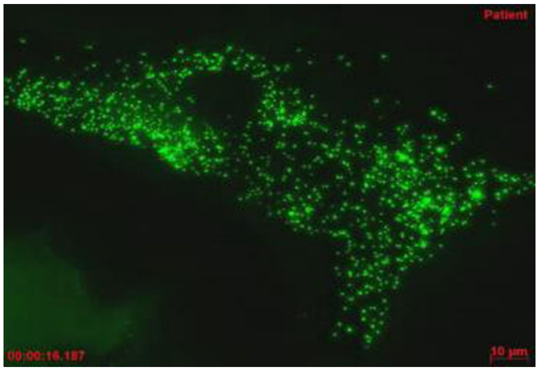

Movie 2. Representative time-lapse movie showing peroxisomes in a HSP patient-derived ONS cell. Example of peroxisomes moving within cells. Compressing the images into this movie format severely reduces image quality. Peroxisomes are green dots. Most show vibratory, Brownian-like motion while some show bursts of rapid, saltatory microtubule-based movement. The movie was captured for duration of 2 minutes at 2 second intervals.
